# Different duration strategies of perioperative antibiotic prophylaxis in adult patients undergoing cardiac surgery: an observational study

**DOI:** 10.1186/s13019-015-0225-x

**Published:** 2015-02-26

**Authors:** Khaled Hamouda, Mehmet Oezkur, Bhanu Sinha, Johannes Hain, Hannah Menkel, Marcus Leistner, Rainer Leyh, Christoph Schimmer

**Affiliations:** 1 Department of Cardiothoracic- and Thoracic Vascular Surgery, University Hospital Würzburg, Oberdürrbacherstraße 6, 97080 Würzburg, Germany; 2 Medical Microbiology, University Medical Center Groningen, Groningen, Netherlands; 3 University of Würzburg, Institute of Mathematics and Informatics, Chair of Mathematics VIII (Statistics), Würzburg, Germany

**Keywords:** Cardiac surgery, Antibiotic prophylaxis, Nosocomial infection

## Abstract

**Background:**

All international guidelines recommend perioperative antibiotic prophylaxis (PAB) should be routinely administered to patients undergoing cardiac surgery. However, the duration of PAB is heterogeneous and controversial.

**Methods:**

Between 01.01.2011 and 31.12.2011, 1096 consecutive cardiac surgery patients were assigned to one of two groups receiving PAB with a second-generation cephalosporin for either 56 h (group I) or 32 h (group II). Patients’ characteristics, intraoperative data, and the in-hospital follow-up were analysed. Primary endpoint was the incidence of surgical site infection (deep and superficial sternal wound-, and vein harvesting site infection; DSWI/SSWI/VHSI). Secondary endpoints were the incidence of respiratory-, and urinary tract infection, as well as the mortality rate.

**Results:**

615/1096 patients (56,1%) were enrolled (group I: n = 283 versus group II: n = 332). There were no significant differences with regard to patient characteristics, comorbidities, and procedure-related variables. No statistically significant differences were demonstrated concerning primary and secondary endpoints. The incidence of DSWI/SSWI/VHSI were 4/283 (1,4%), 5/283 (1,7%), and 1/283 (0,3%) in group I versus 6/332 (1,8%), 9/332 (2,7%), and 3/332 (0,9%) in group II (p = 0,76/0,59/0,63). In univariate analyses female gender, age, peripheral arterial obstructive disease, operating-time, ICU-duration, transfusion, and respiratory insufficiency were determinants for nosocomial infections (all ≤ 0,05). Subgroup analyses of these high-risk patients did not show any differences between the two regimes (all ≥ 0,05).

**Conclusions:**

Reducing the duration of PAB from 56 h to 32 h in adult cardiac surgery patients was not associated with an increase of nosocomial infection rate, but contributes to reduce antibiotic resistance and health care costs.

## Background

The principles of antibiotic prophylaxis are based on (1) the choice of the antimicrobial agent; (2) the timing of the first administered dose, and (3) the duration of the prophylactic regimen [1]. Concerning *the choice* of the antimicrobial agent second-generation cephalosporins in particular have several advantages over other antibiotic choices. They provide broad-spectrum coverage targeting both gram-positive and gram-negative organisms, with good tissue penetration. Furthermore, they have a good safety profile with minimal side effects, and can be tolerated by penicillin allergic patients. However, a disadvantage of cephalosporins is a well-established association with Clostridium difficile infection [2]. Several studies described that there is a trend towards prescribing more second generation cephalosporins [3,4]. Also, the German Paul-Ehrlich-Gesellschaft e.V. (PEG) recommends a second-generation cephalosporin as antibiotic prophylaxis in patients undergoing cardiac surgery [5].

Concerning *the duration* of the prophylactic regimen the data of the international guidelines and recommendations of antibiotic prophylaxis in adult cardiac surgery patients are heterogeneous. The 2011 American College of Cardiology Foundation/American Heart Association guideline for coronary artery bypass graft surgery recommends preoperative antibiotic prophylaxis with additional application for prolonged operations [6,7]. Even the recommendation from the PEG concerning the duration is based only on consensus of the expert panel because the data do not delineate the optimal duration of prophylaxis. The panel suggests the prophylaxis for 24 hours or less as appropriate for cardiothoracic procedures [5]. There is no common recommendation of single dose administration or for longer than a 48-hour regimen. Gorski et al. demonstrated in a nationwide questionnaire was distributed to all German heart surgery centers concerning antibiotic prophylaxis in adult cardiac surgery patients that 100% of all German heart centers use an antibiotic prophylaxis [8]. But the duration strategy of antibiotic prophylaxis in adult cardiac surgery patients varied wildly. 23% use a single-shot prophylaxis, 29% use it for 16 hours, 27% use it for 24 hours, 13% use it for 32 hours, and still 8% use it for 40 hours [8].

Most guidelines *suggest* that prophylaxis for 48 hours or less *may be* appropriate for cardiothoracic procedures [1,5,6,9,10]. Table 1 shows an overview of the different international guidelines regarding the antibiotic prophylaxis for cardiac surgery patients.

**Table 1:** **Guidelines for the duration of antibiotic prophylaxis in cardiac surgery**

	Duration
STS	There is evidence indicating that antibiotic prophylaxis of 48-hours duration is effective. There is some evidence that single-dose prophylaxis or 24-hour prophylaxis may be as effective as 48-hour prophylaxis, but additional studies are necessary before confirming the effectiveness of prophylaxis lasting less than 48 hours. There is no evidence that prophylaxis administered for longer than 48 hours is more effective than a 48-hour regimen.
PEG	The duration is based on consensus of the expert panel because the data do not delineate the optimal duration of prophylaxis. Prophylaxis for 24 hours or less may be appropriate for cardiothoracic procedures.
SIPGWW	The consensus of the workgroup is that administration of prophylaxis for < 24 hours is acceptable and that there is no evidence that providing antimicrobials for longer periods will reduce surgical site infection rates.
ACC/AHA	Data suggest that a 1-day course of intravenous antimicrobials is as efficacious as the traditional 48-hour (or longer) regimen.
ASHP	Prophylaxis for 24 hours or less may be appropriate for cardiothoracic procedures.

*STS* The Society of Thoracic Surgeons, *PEG* Paul-Ehrlich-Gesellschaft e.V., *SIPGWW* Surgical Infection Prevention Guideline Writers Workgroup, *ASHP* American Society of Health-System Pharmacists Commission on Therapeutics.

The incidence of nosocomial infections after cardiac surgery is described with 2,7% [11] up to 26,8% [12] in recent literature. They represent serious complications associated with substantial morbidity and mortality as well as economic burden [5,9,13-15]. Therefore, routine administration of perioperative antibiotic prophylaxis in cardiac surgery patients is well accepted, but the duration for which the antibiotics should be administered is far from settled [2]. There has been a general move towards the use of shorter courses of antibiotics for surgical prophylaxis in order to reduce toxicity, selection of resistant organisms, Clostridium difficile infection and cost [7]. The development of antibiotic-resistant infections has been associated with significantly greater hospital mortality rates compared to similar infections caused by antibiotic-sensitive pathogens [16]. However, cardiac surgery patients leave the operating room with indwelling chest catheters and central venous and arterial lines that can be potential routes for bacterial entry and increase the risk of infection [2].

The purpose of this study was to evaluate the effect of reducing the duration of perioperative antibiotic prophylaxis in adult cardiac surgery patients from 56 hours to a 32 hours use on the incidence of surgical site infections, nosocomial infections, and the mortality rate.

## Methods

### Study design and patient population

This retrospective observational study analyses 1096 cardiac surgical patients consecutively subjected to cardiac surgery between 01.01.2011 and 31.12.201 at the University Hospital Würzburg, Department of thoracic and cardiovascular surgery. Inclusion criteria were defined as follows: Male and female patient aged 18 years or older, heart surgery procedure ± extracorporal circulation (coronary artery bypass grafting ± valve surgery). Exclusion criteria were defined as follows: preoperative signs of infection, history of allergy to the antibiotic to be used in this study, transapical or transfemoral aortic valve implantation, participation in another clinical study. Each patient routinely received perioperative prophylaxis for a fixed period of time with i.v. cefuroxime (1.5 g every 8 hours). Furthermore, all patients were treated with the same glycemic control protocol. In order to improve the quality of medical care we initially reduced the perioperative antibiotic prophylaxis strategy from 56 h towards 32 h duration. Therefore, the patients were divided into two groups according to the timing of surgery. Group I included 283 patients from 01.01.2011 to 30.06.2011 who received 56 h of PAB and group II included 332 patients from 01.07.2011 to 31.12.2011 who received 32 h of PAB. The preoperative, intraoperative, and postoperative protocol for preventing wound infections was not changed during the course of this study.

### Data collection

Medical records were checked for demographic, preoperative, intraoperative and postoperative data and complications. Because of the retrospective design of this study, an ethics votum or signed informed consent was waived. Data were processed and analysed respecting every patient’s anonymity. The corresponding author had full access to all data and had final responsibility for the decision to submit for publication.

### Definition of study variables and End points

All patients were examined once daily up to the time of discharge for wound healing and signs of infection by the treating physicians within the routine clinical course. Preoperatively and on postoperative days 1, 2, 4, and 7 and on the day before discharge, the leucocyte count and C-reactive protein (CRP) were determined. If infection was suspected, these parameters were additionally measured on an ad hoc basis. Surgical wound infection (deep,- and superficial sternal wound infection and vein harvesting site infection), respiratory tract infection, and urinary tract infection were defined according to the guidelines published by the Centers for Disease Control and Prevention [11,17]. Sepsis was defined as the presence of whole body inflammatory state in the presence of a known or suspected infection [5,18,19]. Primary endpoint was the development of microbiologically documented surgical site infection (SSI), like deep sternal wound infection (DSWI), superficial sternal wound infection (SSWI) and vein harvesting site infection (VHSI). Secondary endpoints were the occurrence of nosocomial infection (respiratory tract infection and urinary tract infection) as well as the all-cause mortality rate, including the infection and non-infection related mortality rate. The follow-up time for all patients was until discharge. The mortality was followed-up until 30 days postoperatively. If a patient was readmitted to hospital because of a SSI, the data were involved in the analysis.

### Statistical analysis

Statistical analysis was performed by an independent statistician at the Institute of Mathematics and Informatics, Chair of Mathematics VIII (Statistics), University of Würzburg. The open source software R (version 2.12.1) was used. A p value of ≤ 0,05 was deemed to be statistically significant. For ratio-scaled variables a descriptive overview of the two groups (active drug vs. placebo) was always prepared. The two groups were then analysed with the Mann–Whitney *U* test for significant differences. A group overview was also prepared for nominal-scaled variables. To determine differences in these values, the Chi-square test of independence was performed. If the variable was binomial (e.g. gender) Fisher’s exact test was applied and the odds ratio with a 95% confidence interval was calculated. The subgroup analysis was performed with Fisher's exact test or the Mann–Whitney *U* test according to the scale type of the corresponding measurement. For all tests in the subgroup analysis the Benjamini-Hochberg correction of the significance level was conducted. To compare the two estimates of the two groups, a log-rank test was done. The data were processed and analysed while preserving the patient’s anonymity.

## Results

**Figure 1:**
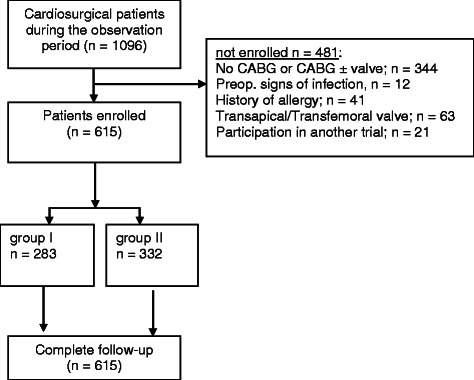
**Trial profile.**

615/1096 (56,1%) of the screened patients were included in the study analysis. 481/1096 (43,9%) patients could not be evaluated, because of the above mentioned inclusion and/or exclusion criteria (Figure 1). 283/615 (46%) patients were assigned to group I (56 h of PAB) and 332/615 (54%) to group II (32 h of PAB).

There were no significant differences with regard to patient characteristics, comorbidities, and procedure-related variables (Table 2).

**Table 2:** **Patient characteristics**, **comorbidities**, **and procedure**-**related variables**

	All	Group I	Group II	p-value
	n = 615	n = 283	n = 332	
*Sex*				0,85
• Men; n (%)	464 (75,4)	215 (75,9)	249 (75)	
• Female; n (%)	151 (24,6)	68 (24,1)	83 (25)	
Age (years)	68,7 ± 9,8	69,2 ± 9,7	68,3 ± 9,9	0,27
BMI (kg/m^2^)	28,3 ± 4,2	28,3 ± 4,0	28,3 ± 4,4	0,80
Diabetes mellitus; n (%)	181 (29,4)	88 (31)	93 (28)	0,89
COPD; n (%)	82 (13,3)	34 (12)	48 (14,4)	0,22
PAOD; n (%)	61 (9,9)	23 (8,1)	38 (11,4)	0,18
Creatinine (mg/dL)	1,0 ± 0,3	1,0 ± 0,2	1,0 ± 0,3	0,44
Dialyse; n (%)	13 (2,1)	7 (2,4)	6 (1,8)	0,59
EF < 30%; n (%)	53 (8,6)	23 (8,1)	30 (9,0)	0,93
*Resucitation*				0,60
• No; n (%)	592 (96,2)	275 (97,1)	317 (95,4)	
• <48 h	13 (2,1)	4 (1,4)	9 (2,7)	
• <21 days	9 (1,4)	4 (1,4)	5 (1,5)	
• >21 days	1 (0,1)	0 (0)	0 (0)	
PMV; n (%)	10 (1,6)	5 (1,7)	5 (1,5)	1,00
IABP preoperative; n (%)	111 (18)	46 (16,2)	65 (19,5)	0,30
*State of operation; n (%)*				0,79
• Elective	416 (67,6)	197 (69,6)	219 (65,9)	
• Urgent	138 (22,4)	59 (20,8)	79 (23,7)	
• Emergency	53 (8,6)	23 (8,1)	30 (9,0)	
• Resuscitation	8 (1,3)	4 (1,4)	4 (1,2)	
*Type of procedure; n (%)*				0,30
• isolated CABG	550 (89,4)	249 (87,9)	301 (90,6)	
• CABG ± valve surgery	65 (10,6)	34 (12,1)	31 (9,4)	
*Procedure related variables*				
• Operating time (min)	218 ± 55	219 ± 58	218 ± 53	0,88
• Bypass time (min)	95 ± 32	97 ± 34	94 ± 30	0,49
• Clamping time (min)	69 ± 25	70 ± 26	68 ± 24	0,38
OPCAB; n (%)	36 (5,8)	18 (6,3)	18 (5,4)	0,41
ICU duration (days)	2,9 ± 2,6	2,9 ± 2,5	3,0 ± 2,6	0,66
*Mobilisation; n (%)*				0,44
1. pod	116 (18,8)	51 (18,0)	65 (19,5)	
2. pod	255 (41,4)	130 (45,9)	125 (37,6)	
3. pod	178 (28,9)	79 (27,9)	99 (29,8)	
Not mobilized	30 (10,9)	13 (8,2)	17 (12,9)	
Transfusion of RBC (n)	1,5 ± 3,3	1,5 ± 3,1	1,5 ± 3,6	0,87
Resuscitation; n (%)	21 (3,4)	10 (3,5)	11 (3,3)	1,00
*Reintubation; n (%)*	32 (5,2)	16 (5,6)	16 (4,8)	0,70
• Tracheotomy	5 (0,8)	2 (0,7)	3 (0,9)	
TPS; n (%)	26 (4,2)	13 (4,5)	13 (3,9)	0,92
Revision; n (%)	38 (6,1)	21 (7,4)	17 (5,1)	0,18
Periop. MI; n (%)	5 (0,8)	1 (0,3)	4 (1,2)	0,38

*BMI* Body Mass Index, *COPD* chronic obstructive pulmonary disease, *PAOD* peripheral arterial obstructive disease, *EF* ejection fraction, *PMV* preoperative mechanical ventilation, *IABP* intraaortic balloon pulsation, *CABG* coronary artery bypass grafting, *OPCAB* off-pump coronary artery bypass, *ICU* intensive care unit, *pod* postoperative day, *RBC* red blood concentrate, *TPS* transitory psychotic syndrome, *Periop. MI* perioperative myocardial infarction.

Regarding every single microbiological documented infection 97 hits could be found in 615 patients (15,8%). The incidence of deep sternal wound infection, superficial sternal wound infection, and vein harvesting site infection were 4/283 (1,4%), 5/283 (1,7%), and 1/283 (0,3%) in group I versus 6/332 (1,8%), 9/332 (2,7%), and 3/332 (0,9%) in group II (p = 0,76/0, 59/0,63), respectively. Also, no statistically significant differences were demonstrated concerning secondary end-points. The all-cause mortality after 30 days was 23/615 (3,7%) patients (group I, 2,4% vs. group II, 4,8%, p = 0,14). The infection related mortality rate was 2/283 (0,7%) patients in group I versus 4/332 (1,2%) patients in group II (Table 3).

**Table 3:** **Primary and secondary endpoints**

	All	Group I	Group II	p-value
	n = 615	n = 283	n = 332	
**Primary endpoints**				
DSWI; n (%)	10 (1,6)	4 (1,4)	6 (1,8)	0,76
SSWI; n (%)	14 (2,2)	5 (1,7)	9 (2,7)	0,59
VHSI; n (%)	4 (0,6)	1 (0,3)	3 (0,9)	0,63
**Secondary endpoints**				
Respiratory tract infection; n (%)	23 (3,7)	12 (4,2)	11 (3,3)	0,67
Urinary tract infection; n (%)	46 (7,4)	26 (9,1)	20 (6,0)	0,17
All-cause mortality; n (%)	23 (3,7)	7 (2,4)	16 (4,8)	0,14
• Mortality due to infection	6 (0,98)	2 (0,71)	4 (1,20)	
• Mortality due to other cause	17 (2,76)	6 (2,12)	11 (3,31)	

*DSWI* deep sternal wound infection, *SSWI* superficial sternal wound infection, *VHSI* vein harvesting site infection.

In this observational study, we were able to determine why these patients were more likely to receive nosocomial infections: female (p = 0,02), age > 80 years (p = 0,05), peripheral arterial obstructive disease (p = 0,02), operating time > 240 min (p = 0,01), ICU duration > 3 days (p = 0,01), transfusion of > 5 RBC (p = 0,02), and respiratory insufficiency (p = 0,01) (Table 4).

**Table 4:** **Univariate analyses**

	Infection	No-Infection	p-value
*Sex; n (%)*			0,02
• Men	58 (12,5)	406 (87,5)	
• Female	31 (20,5)	120 (79,5)	
*Age; n (%)*			0,05
• >80 years	18 (23,7)	58 (76,3)	
• <80 years	71 (13,2)	468 (86,8)	
*BMI; n (%)*			0,25
• >30 kg/m^2^	26 (13,8)	162 (86,2)	
• <30 kg/m^2^	63 (14,8)	364 (85,2)	
*Diabetes mellitus; n (%)*			0,33
• Yes	32 (17,1)	149 (82,3)	
• No	57 (13,2)	377 (86,9)	
*PAOD; n (%)*			0,02
• Yes	38 (19,3)	159 (80,7)	
• No	12,2 (51)	367 (87,8)	
*COPD; n (%)*			0,17
• Yes	17 (20,7)	65 (79,3)	
• No	72 (13,5)	461 (86,5)	
*Dialyse; n (%)*			1,00
• Yes	2 (15,4)	11 (84,6)	
• No	87 (14,5)	515 (85,5)	
*State of operation; n (%)*			0,82
• Elective	63 (15,1)	353 (84,9)	
• Urgent	17 (12,3)	121 (87,7)	
• Emergency	8 (15,1)	45 (84,9)	
• Resuscitation	1 (12,5)	7 (87,5)	
*Operating time; n (%)*			0,01
• >240 min	43 (20,6)	166 (79,4)	
• <240 min	47 (11,3)	360 (88,7)	
*Bypass time; n (%)*			0,16
• >90 min	51 (16,7)	254 (83,3)	
• <90 min	31 (11,2)	246 (88,8)	
*Clamping time; n (%)*			0,51
• >60 min	55 (15,5)	299 (84,5)	
• <60 min	25 (11,8)	186 (88,2)	
*IABP postoperative; n (%)*			0,23
• Yes	9 (14,8)	52 (85,2)	
• No	80 (14,4)	474 (85,6)	
*ICU duration; n (%)*			0,01
• <3 days	36 (10,5)	309 (89,6)	
• >3 days	53 (19,7)	217 (80,4)	
*Perioperative MI; n (%)*			0,64
• Yes	0 (0)	5 (100)	
• No	89 (14,5)	521 (85,4)	
*IABP; n (%)*			0,23
• Yes	9 (14,8)	52 (85,2)	
• No	80 (14,4)	474 (85,6)	
*Transfusion of > 5 RBC; n (%)*			0,02
• Yes	2,8 (23)	70 (75,3)	
• No	66 (12,7)	456 (87,4)	
*Respiratory insufficiency; n (%)*			0,01
• Yes	8 (57,1)	6 (42,9)	
• No	79 (13,6)	503 (86,4)	
*Transitory psychotic syndrome; n (%)*			0,34
• Yes	5 (25)	15 (75)	
• No	84 (14)	511 (86)	
*Mortality after 30 days; n (%)*			0,11
• Yes	6 (26)	17 (74)	
• o	83 (14)	508 (86)	

*BMI* Body Mass Index, *PAOD* peripheral arterial obstructive disease, *COPD* chronic obstructive pulmonary disease, *IABP* intraaortic balloon pulsation, *ICU* intensive care unit, Periop. *MI* perioperative myocardial infarction, *RBC* red blood concentrate.

Analysing these patients with an increased risk for nosocomial infection relating to the two different antibiotic duration regimes (group I versus group II) no statistically significant difference could be demonstrated (Table 5).

**Table 5:** **Subgroup analyses**

Risk factors	Group I(56 h)	Group II(32 h)	p-value
Female	23,5%	22,3%	0,82
Age > 80 years	23,7%	23,7%	1,00
PAOD	16,9%	25,3%	0,20
Operating time 240 min	22,8%	20,3%	0,63
ICU duration < 3 days	20,5%	21,0%	1,00
Transfusion of > 5 RBC	26,5%	29,6%	0,91
Respiratory insufficiency	62,5%	66,7%	1,00

*PAOD* peripheral arterial obstructive disease, *ICU* intensive care unit, *RBC* red blood concentrate.

## Discussion

The majority of published evidence in general surgery demonstrates that antimicrobial prophylaxis after wound closure is unnecessary, and most studies comparing single-dose prophylaxis with multiple-dose prophylaxis have not shown benefit of additional doses [10]. But there are several reasons why prolonged (24 – 48 hours) prophylactic regimens should be used in *cardiac surgery*, such as cardiopulmonary bypass and systemic cooling for myocardial protection, invasive devices remaining after surgery, high risk of bleeding requiring blood transfusion and re-exploration, and delayed extubation after surgery. Furthermore, there are few data on the pharmacokinetics of antibiotics during cardiopulmonary bypass, and therefore dosing regimens are often based on historical practice [7]. Besides these reasons, results from other trials in cardiac surgery described that increasing the duration of antibiotic prophylaxis in cardiac surgery patients did not result in a significant decrease in surgical site infections [3,4,10,20-23]. Therefore, it is generally accepted that short-term perioperative antibiotic prophylaxis is as efficacious in preventing postoperative complications as longer-term prophylaxis. However, the optimal duration of antibiotic prophylaxis in cardiac surgery is controversial [21]. Recommendations for perioperative antibiotic prophylaxis in cardiac surgery vary, ranging from single infusion of antibiotics [3,22] to durations up to 72 hours [21,24].

### Comparison of single-versus multiple-dose perioperative antibiotic prophylaxis

Nooyen et al. described in a prospective randomised comparison study (n = 844 patients) that a single dose of cefuroxime is as effective as a three-day course in the prevention of wound infection (sternal site infection; p = 0,35 and donor site infection; p = 0,41) [3]. This study showed many exclusion criteria and the power of this study is too low to draw any conclusion out of it. Even Bucknell et al. showed in a non-randomized trial with 353 consecutive patients that a single-dose antimicrobial prophylaxis (cefazolin) is as effective as a 48-hour regimen. There was no significant difference in rate of infection between the two groups (p = 0,89) [22]. On the other hand, Tamayo et al. showed in a random, prospective, clinical study included 838 adult patients that single-dose-cefazolin is associated with a higher surgical site infection rate than the 24 hour multiple-dose cefazolin regimen (8,3% vs 3,6%; p = 0,00) [25]. The follow-up period of this study was 12 months postoperatively, the follow-up period of the studies mentioned above by Nooyen et al. and Bucknell et al. evaluated the sternal site infection only over 7 postoperative days. This limitation is important because it is well known that sternal infections usually manifest themselves from the second postoperative week onward [23].

### Comparison of different multiple-dose perioperative antibiotic prophylaxis

The results of the present study support the conclusion of Gupta et al. [2]. They compared 235 adult patients undergoing elective cardiac surgery in a randomized double blind study. The groups received prophylactic antibiotic therapy for either 48 hours or 72 hours. The results showed that 48 hours prophylactic antibiotic therapy is at least as effective as a 72 hours regimen in relation to surgical site infection (p > 0,05), but prevents the potential of causing an increase in antibiotic resistance [24]. Furthermore, the literature contains 2 meta-analysis on patients undergoing cardiac surgery and the duration of perioperative antibiotic prophylaxis [21,26]. The first meta-analysis by Mertz et al. included 7893 patients (including 12 studies) focusing on the risk of sternal surgical site infections between short-term antibiotic prophylaxis (*<24 hours*) versus longer-term antibiotic prophylaxis (*≥24 hours*) among adult patients undergoing open heart surgery. The authors found a reduced risk of sternal surgical site infection by 38% (p = 0,01) in patients with longer-term antibiotic prophylaxis. Therefore, they concluded that antibiotic prophylaxis of *> 24 hours* may be more efficacious in preventing sternal SSIs compared to shorter regimens. Similar to our results these studies do not found any significant differences in mortality and overall rate of infection. The findings however are limited by the heterogeneity of antibiotic regimens used and the risk of bias in the published studies [21]. The second study published on this topic was carried out by Lador et al. They reported of 23 randomized controlled trials and stated that in trials comparing different durations, prophylaxis of *≤ 24 h post-operation* led to higher rates of DSWI, any sternal SSI, surgical interventions for SSI and endocarditis compared with longer duration prophylaxis. But there was no advantage of regimens lasting *> 48 hours* post-operation. However, the authors indicate that prolonging prophylaxis include the induction of resistant bacteria that may affect the individual patient and surrounding patients. This was mostly not assessed in existing trials [26]. But the trials included in these two meta-analyses were performed with different antibiotic regimens and were pooled for the analyses. Therefore, they relay on studies comparing different antibiotic regimens [4].

Fowler et al. identified and validated a model (n = 331429 CABG patients) that identify patients undergoing cardiac surgery who are at high risk for major infection (Age, BMI, Diabetes, Renal failure, Congestive heart failure, Peripheral vascular disease, Female gender, Chronic lung disease, Cardiogenic shock, Myocardial infarction, Concomitant surgery, Perfusion time 100 to 300 minutes, and Intra-aortic balloon pump [15]. These results were similar to our findings. These high-risk patients may be targeted for perioperative intervention strategies to reduce rates of major infection [15]. On the basis of these findings, we intend a prospective randomized trial calculating the duration of perioperative antibiotic prophylaxis depending on this individualized scoring system for cardiac surgery patients (single dose versus 24 hours). Limitations of this study are the following facts: First, we performed a retrospective observational single-center study over a time period of one year with consecutive patients rather than a prospective randomised multicenter trial. Second, the small number of patients (n = 615) did not provide sufficiently the power to analyse the effect of reducing the perioperative antibiotic prophylaxis.

## Conclusions

On the basis of the international literature, the different guidelines and the results obtained in this observational study, reducing the duration of perioperative antibiotic prophylaxis from 56 h to 32 h in adult cardiac surgery patients does not increase the rate of surgical site infection, nosocomial infection and the mortality rate, but it contributes to reduce antibiotic resistance and health care costs.

## Abbreviations

PEG: German Paul-Ehrlich-Gesellschaft e.V
PAB: Perioperative antibiotic prophylaxis
CRP C: Reactive protein
SSI: Surgical site infection
DSWI: Deep sternal wound infection
SSWI: Superficial sternal wound infection
VHSI: Vein harvesting site infection
ICU: Intensive care unit
